# Arylamine N-acetyltransferase 1 protects against reactive oxygen species during glucose starvation: Role in the regulation of p53 stability

**DOI:** 10.1371/journal.pone.0193560

**Published:** 2018-03-08

**Authors:** LiLi Wang, Rodney F. Minchin, Neville J. Butcher

**Affiliations:** Laboratory for Molecular and Cellular Pharmacology, School of Biomedical Sciences, University of Queensland, Brisbane, Australia; Universite Paris Diderot, FRANCE

## Abstract

Human arylamine N-acetyltransferase 1 (NAT1) has been associated with cancer cell growth and invasion, but the underlying molecular mechanisms remain unknown. NAT1 is located on the short arm of chromosome 8 (8p21), a region that is commonly deleted in colon cancer. Previously, it was reported that HT-29 colon cancer cells, which have a large deletion at 8p21-22, show marked morphological changes, increased E-cadherin expression and altered cell-cell contact inhibition following down-regulation of NAT1 with shRNA. By contrast, no effects on growth were observed in HeLa cells. In the present study, cellular changes following knockout of NAT1 with CRISPR/Cas9 in HT-29 and HeLa cells were compared in the presence and absence of glucose. Cell growth decreased in both cell-lines during glucose starvation, but it was enhanced in HT-29 cells following NAT1 deletion. This was due to an increase in ROS production that induced cell apoptosis. Both ROS production and cell death were prevented by the glutathione precursor N-acetylcysteine. NAT1 knockout also resulted in a loss of the gain-of-function p53 protein in HT-29 cells. When p53 expression was inhibited with siRNA in parental HT-29 cells, ROS production and apoptosis increased to levels seen in the NAT1 knockout cells. The loss of p53 may explain the decreased colony formation and increased contact inhibition previously reported following NAT1 down-regulation in these cells. In conclusion, NAT1 is important in maintaining intracellular ROS, especially during glucose starvation, by stabilizing gain-of-function p53 in HT-29 cells. These results suggest that NAT1 may be a novel target to decrease intracellular gain-of -function p53.

## Introduction

The arylamine N-acetyltransferases are a family of Phase II drug metabolizing enzymes that utilise acetyl coenzyme A to acetylate hydrazines, aromatic amines and heterocyclic amines [1]. In humans, there are two closely related enzymes, NAT1 and NAT2 which share an 87% amino acid sequence identity but have different substrate specificities. NAT1 is widely expressed in adult and fetal tissues while NAT2 is found primarily in the liver, intestines and colon [2]. Several recent studies suggest that NAT1 can protect cells during nutrient deprivation. This was seen when HT-29 cells were grown continuously for 6 days without a change in medium during which inhibition of NAT1 significantly reduced cell survival [3]. Similarly, when methionine was removed from the medium, cells died more rapidly in the absence of NAT1 [4]. Over-expression of NAT1 in HB4a cells protected them from growth inhibition in low serum conditions [5]. *In vivo*, tumors from NAT1 knockdown HT-29 cells developed significantly more slowly than those from the parental cell-line [6] while NAT1 inhibition in MDA-MB-231 cells slowed growth *in vitro* and inhibited *in vivo* metastasis to the lungs [7]. Knockdown of NAT1 promotes a more epithelial phenotype with up-regulation of E-cadherin, cell-cell contact inhibition and loss of filopodia [3, 6, 7].

These effects of NAT1 on growth and metastatic potential are supported by observations in cancer patients. NAT1 is significantly elevated and correlates with epithelial to mesenchymal activation in breast cancer bone metastasis [8]. Moreover, metastatic disease retains the level of NAT1 expression seen in primary tumors, at least for breast cancers [2]. In melanoma, NAT1 expression increased as tissue progressed from benign to vertical growth and then metastatic disease, suggesting high levels of NAT1 are associated with a more aggressive phenotype [9].

There is now growing evidence that NAT1 has an important cellular function. However, the exact mechanisms of action for the enzyme in these different cellular processes remain unknown. Because NAT1 is genetically variant [10, 11], patients with high NAT1 expression may be at greater risk of aggressive cancers compared to those with low expression. This variation in human NAT1 emphasises the need to better understand the role of NAT1 in cell function. Previous studies have shown that the effects of NAT1 inhibition on growth is cell-dependent. In colon carcinoma HT-29 cells, inhibition of NAT1 slowed growth and inhibited colony formation in soft agar [3]. By contrast, NAT1 inhibition had no effect on growth in HeLa cells [4]. An important difference between these two transformed cell-lines is p53. While HeLa cells express a wild-type p53, HT-29 cells harbor a R^273^H mutation that results in gain-of-function (see p53 database and references therein http://p53/free.fr).

In the present study, the effects of NAT1 deletion in these two cell-lines have been compared in an attempt to identify potential pathways affected by the enzyme. A CRISPR/Cas9 approach was employed to delete NAT1 and the resulting lines were used to evaluate the effects of NAT1 on cell survival under normal and nutrient-deprived conditions.

## Materials and methods

### Cell culture

All cell-lines were purchased from ATCC and were cultured in RPMI 1640 medium supplemented with 10% FBS (Hyclone), 2 mM L-glutamine, and 100 units/ml penicillin/streptomycin (Thermo Fisher Scientific) at 37°C in a humidified atmosphere of 5% CO_2_. Standard RPMI 1640 medium contains 10 mM glucose. For experiments in low glucose, RPMI 1640 minus glucose medium was supplemented with 1 mM glucose (Thermo Fisher Scientific). For siRNA transfections, 20 nM siRNA (Origene) was used with Lipofectamine RNAiMAX reagent (Invitrogen) according to the manufacturer's instructions. A scrambled sequence was used as a control.

### CRISPR/Cas9 knockout of NAT1

The NAT1 gene was disrupted using a human NAT1 gene knockout CRISPR/Cas9 kit (OriGene Technologies, KN221042) according to the manufacturer’s instructions. The kit contained two gRNA vectors with predesigned NAT1 target sequences (g1 5’-GAATTGGCTATAAGAAGTCT-3’ and g2 5’-GTCTAGGAACAAATTGGACT-3’), a negative scramble gRNA, and a donor vector containing a GFP/puromycin selection cassette flanked on either side by sequences homologous to the NAT1 gene. Briefly, cells were co-transfected with equal amounts of gRNA (0.5 μg) and donor vectors (0.5 μg) using Lipofectamine 2000 (Thermo Fisher Scientific). Transfected cells then underwent 7 rounds of 1:10 passages prior to selection with 0.5 μg/ml puromycin. Following puromycin treatment, single colonies were selected, expanded, and then screened for NAT1 activity as previously described [12]. NAT1 knockout was verified by PCR of genomic DNA and Western blot for NAT1 protein (S1 Fig).

### Cell proliferation assay

Cell proliferation was measured using a CyQUANT NF cell proliferation assay kit (Thermo Fisher Scientific). Cells were seeded at a density of 5000 cells per well in 96-well microplates for 5 h in RPMI 1640 medium. The medium was then changed to either high glucose (10 mM) or low glucose (1 mM) in RPMI 1640. At the indicated times, medium was removed from cells and replaced with 50 μl dye reagent diluted in HBSS. After a 30 min incubation at 37 ^o^C, fluorescence was measured using a microplate reader (excitation = 485 nm, emission = 530 nm).

### Glucose uptake

Glucose uptake was quantified using 2-(N-(7-nitrobenz-2-oxa-1,3-diazol-4-yl)amino)-2-deoxyglucose (2-NBDG, Cayman Chemical), a fluorescent-labelled glucose analog, according to published procedures [13].

### Glucose concentration measurements

Cells were seeded at a density of 5000 cells per well in a 96 well plate for 5 h in RPMI 1640. Medium was changed to 1 mM glucose and the cells were grown for 2 days. The medium was collected each day and glucose was measured using the Abcam ab169559 kit.

### Detection of apoptosis

Apoptosis was measured by annexin V staining using a Muse cell analyzer (Merck). Briefly, cells were grown overnight in RPMI 1640 medium containing either 10 mM or 1 mM glucose. They were then trypsinized, washed with PBS and stained with annexin V reagent (Merck) for 20 min and analysed immediately.

### Cellular ROS measurement

Cellular reactive oxygen species (ROS) was quantified by flow cytometry using the DCFDA cellular ROS detection assay kit (Abcam, ab113851) according to the manufacturer’s instructions. Briefly, cells were grown overnight in either normal (10 mM) or low (1 mM) glucose. They were then trypsinized, resuspended in RPMI 1640 medium and stained with DCFDA for 30 min at 37 ^o^C. Analysis was performed using a FACSCanto flow cytometer (BD Bioscience). For some experiments, cells were treated with 10 mM N-acetylcysteine (Sigma Aldrich) overnight prior to ROS measurement.

### Western blotting

Cells were lysed in RIPA buffer (50 mM Tris, pH 8.0, 150 mM NaCl, 1% NP-40, 0.5% sodium deoxycholate, 0.1% SDS) containing protease and phosphatase inhibitor cocktails (Sigma-Aldrich) or boiled directly in Laemmli’s buffer. Proteins were separated by SDS-PAGE and then transferred onto nitrocellulose membranes. After blocking with 5% skim milk in PBST, membranes were incubated with primary antibodies overnight (Cell Signaling Technology; anti-α-tubulin #3873 at 1:2000, anti-β-actin #3700 at 1:2000 and anti-p53 #2524 at 1:1000 dilutions.) followed by HRP-conjugated secondary antibodies (Jackson ImmunoResearch) for 1 h at room temperature. Detection was by ECL Plus (PerkinElmer) and a Kodak Image Station 4000s pro. Quantification was performed by densitometry using ImageJ software.

### Statistical analysis

All data are expressed as mean ± sem. Multiple comparisons were performed by ANOVA using Dunnett’s test for multiple comparisons, while comparisons between 2 groups were performed by Student’s *t*-test. *P*-values of 0.05 or less were considered significant.

## Results

### Loss of NAT1 suppresses cancer cell proliferation and increases apoptosis during glucose starvation

**Fig 1:**
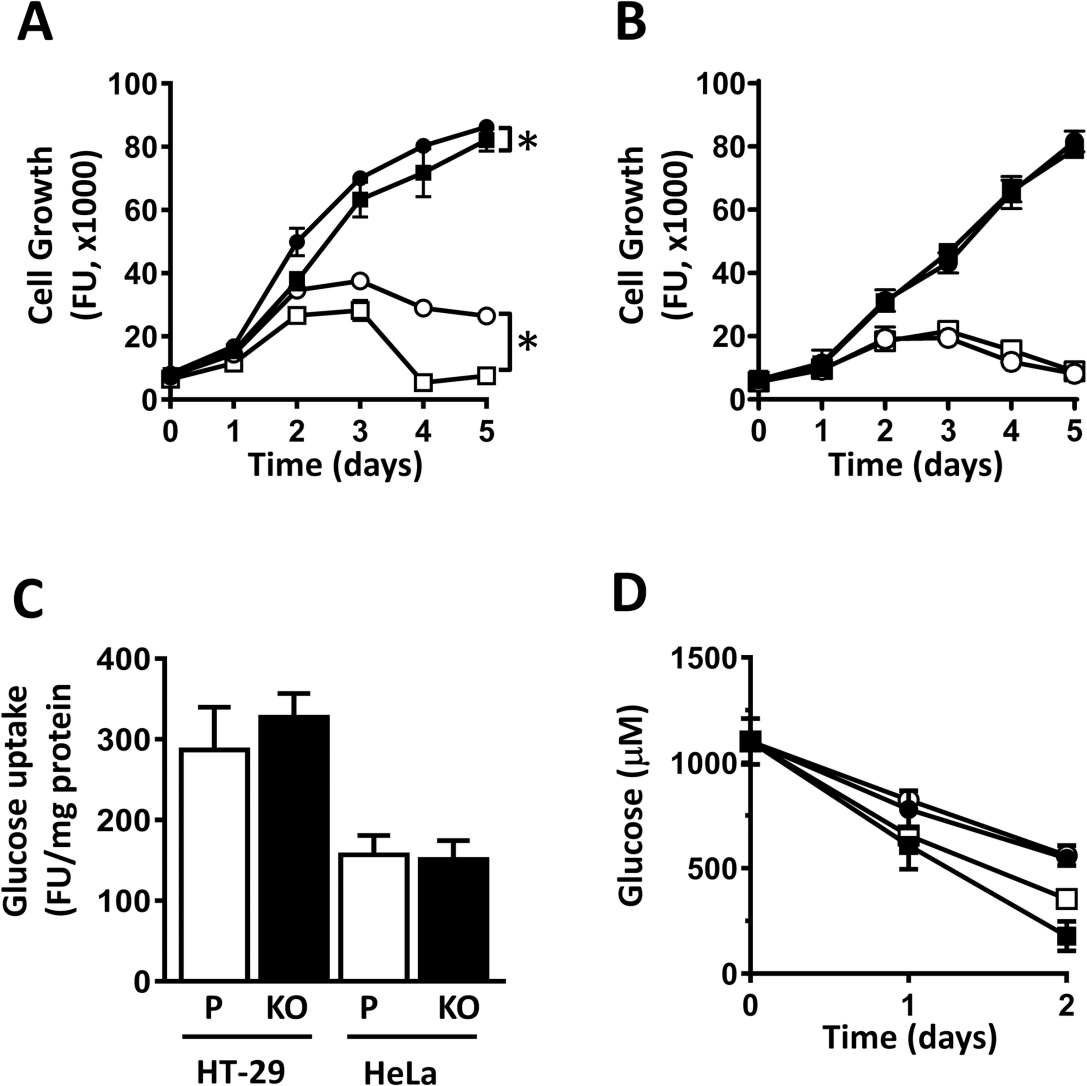
Effect of NAT1 knockout on cell growth in normal and low glucose. (A) HT-29 cell growth in 10 mM glucose (closed symbols) and 1 mM glucose (open symbols) over 5 days without change of media. Parental cells are shown as circles while NAT1 deleted cells are shown as squares. Data are mean ± sem, n = 4. Asterisk indicates significant difference by two-way ANOVA. (B) The same growth conditions as in A for HeLa cells. (C) Glucose uptake in Parental (P) and NAT1 knockout (KO) cells. Data are mean ± sem, n = 5. (D) Glucose concentrations in the media over 2 days of culture for HT-29 cells (squares) and HeLa cells (circles). Parental cells are closed symbols while NAT1 knockout cells are open symbols. Data are mean ± sem, n = 3.

To assess the effect of NAT1 knockout on cell growth, the gene was deleted using CRISPR/Cas9. The resulting lines were validated by the absence of mRNA using RT-PCR, the absence of protein by Western blot and the loss of enzyme activity (S1 Fig). The growth of HT-29 and HeLa cells over 3 days was then measured in normal (10 mM) and low (1 mM) glucose conditions. The growth rate of HT-29 cells was significantly decreased following NAT1 knockout and the effect was more pronounced in low glucose (Fig 1A). By contrast, no effect following NAT1 deletion was seen with the HeLa cells (Fig 1B). The different rates of proliferation following NAT1 deletion was not due to changes in glucose uptake (Fig 1C) or glucose utilization (Fig 1D), which were unaltered in both cell-lines.

**Fig 2:**
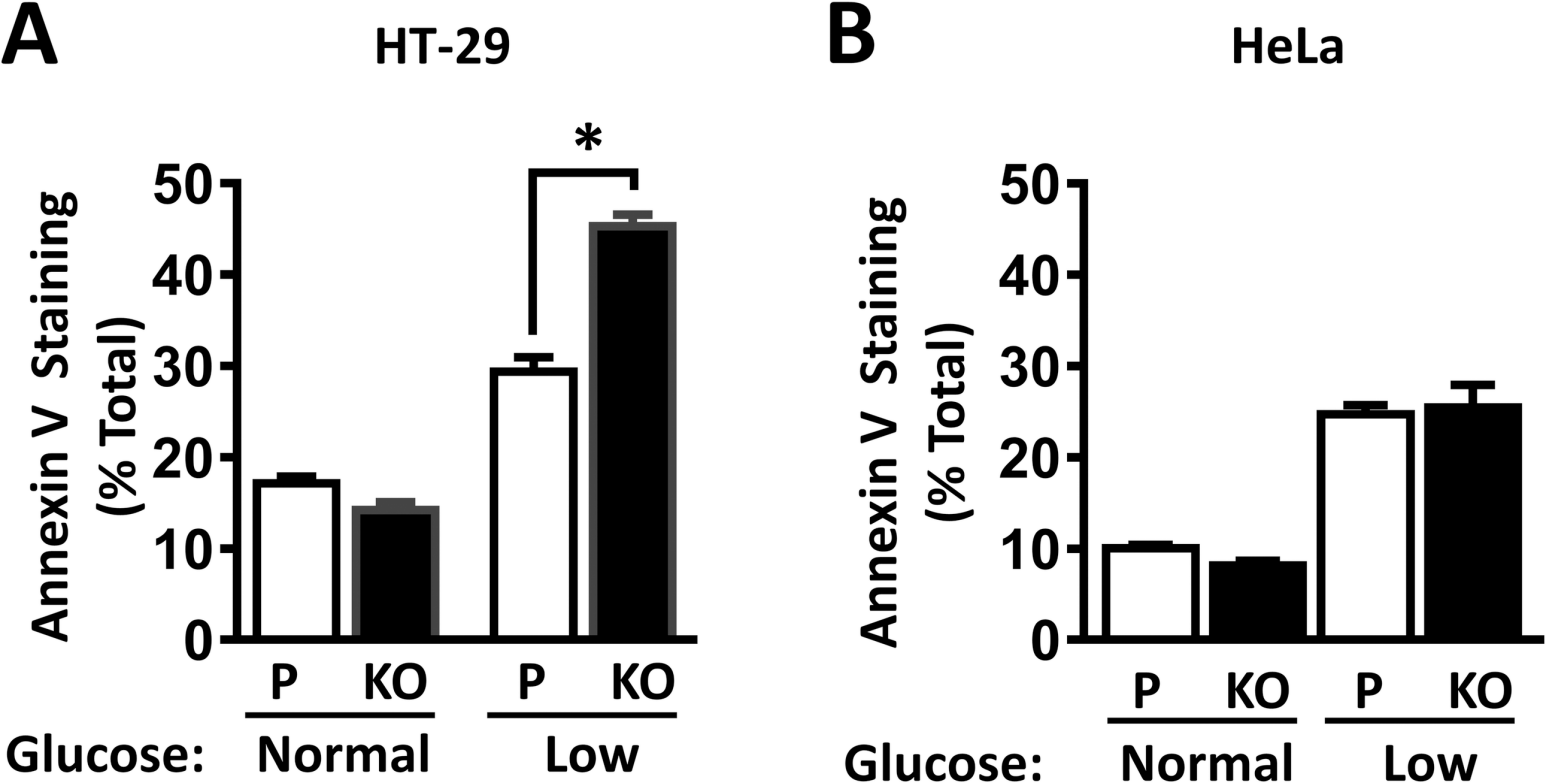
Effect of NAT1 knockout on cell apoptosis. HT-29 (A) and HeLa (B) cells were grown overnight in normal (10 mM) or low (1 mM) glucose and apoptosis was measured by annexin V staining. P = Parental cells; KO = NAT1 knockout cells. Data are mean ± sem, n = 3. Asterisk indicates significant difference by one-way ANOVA.

To identify the reason for the decrease in cell proliferation, the effect of NAT1 knockout on apoptosis was measured over 24 h with glucose starvation. In both HT-29 and HeLa cells, there was no difference in the percent of apoptotic cells grown in high glucose with or without NAT1 (Fig 2A and 2B). In low glucose, the number of apoptotic cells significantly increased for both cell-lines. However, NAT1 deletion further enhanced apoptosis in the HT-29 cells but had no effect in the HeLa cells. These results suggest that the differences in growth over time seen in Fig 1 are due to an increase in apoptosis and that NAT1 enhances apoptosis in a cell-dependent manner.

### Increased cell death following NAT1 knockout is due to ROS generation

**Fig 3:**
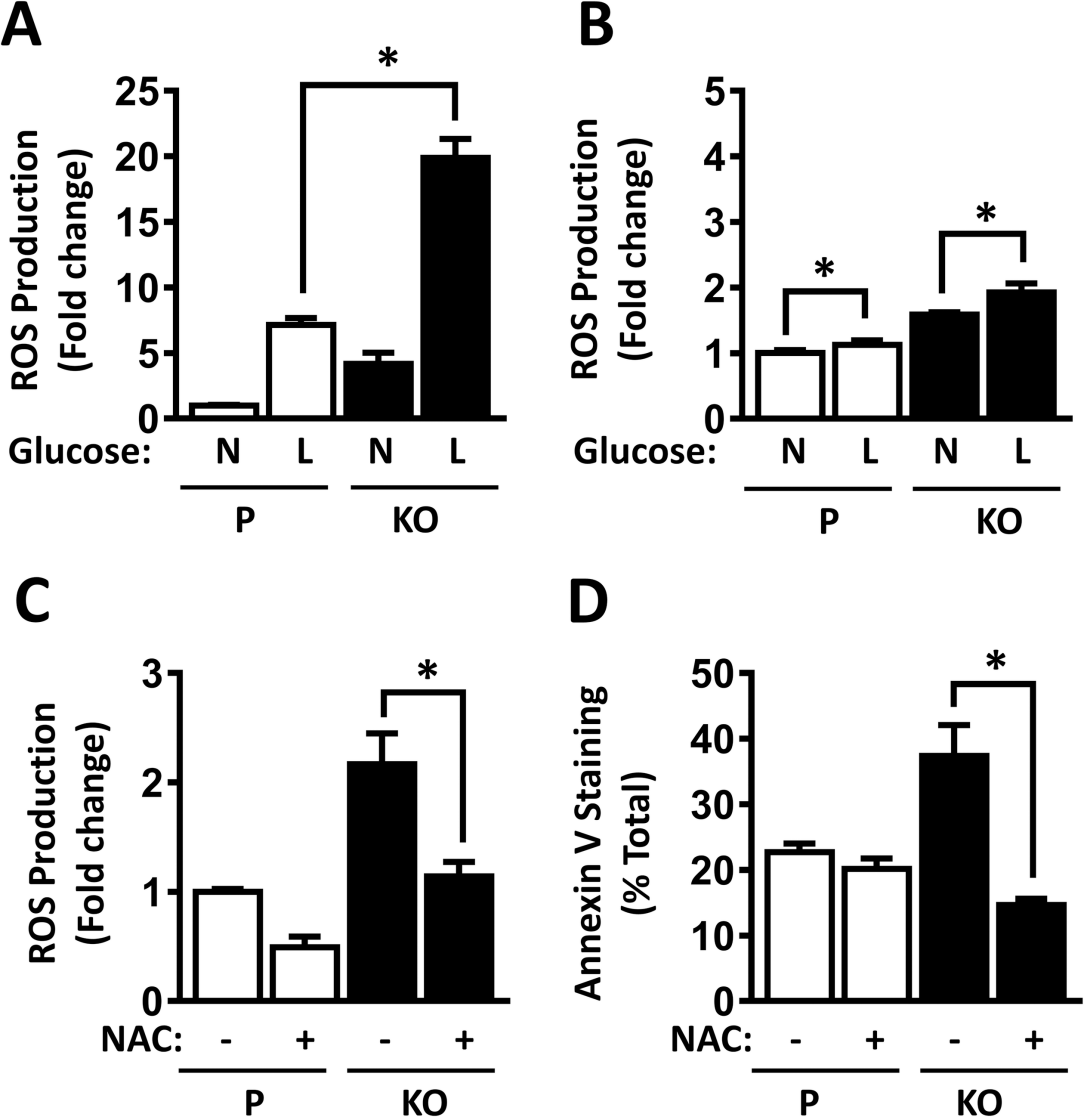
Effect of NAT1 knockout on the production of reactive oxygen species (ROS). Cells were grown in the presence of normal (N: 10 mM) or low (L: 1 mM) glucose for 24 h. Open bars are Parental cells (P) and closed bars are NAT1 knockout cells (KO). Data are expressed as mean ± sem, n = 3. Asterisk indicates significant difference by one-way ANOVA. (A) ROS production in HT-29 cells, expressed as fold change compared to Parental cells in the presence of normal glucose. (B) ROS production in HeLa cells. (C) Effect of N-acetylcysteine (NAC) on ROS production in HT-29 cells grown in low glucose (1 mM) for 24 h. (D) Effect of NAC on apoptosis in HT-29 cells grown in low glucose (1 mM) for 24 h.

Cells exposed to low glucose (1 mM) increase their production of ROS, which can act as regulators of intracellular signaling at low concentrations or induce apoptosis at high concentrations [14, 15]. To determine whether the increase in cell death in the HT-29 cells following NAT1 deletion was due to oxidative stress, ROS were quantified in the parental and NAT1 knockout cells. Glucose starvation significantly increased ROS production (Fig 3A). However, the increase was much greater following NAT1 deletion. By contrast, ROS production was only slightly increased in HeLa cells (Fig 3B). Treatment of the HT-29 cells with the glutathione precursor and ROS scavenger N-acetylcysteine (NAC) decreased ROS levels in both the parental and NAT1 knockout cells (Fig 3C). NAC also reversed the increase in apoptosis seen following glucose starvation (Fig 3D). The data show that low glucose increases ROS production and apoptosis in HT-29 cells. In addition, NAT1 deletion enhances both of these events.

ROS has been reported to inhibit the arylamine N-acetyltransferases in human cells by oxidative attack at the active site cysteine [16]. Following growth of parental HT-29 and HeLa cells in low glucose for 24 h, NAT1 activity decreased by 23% and 51%, respectively. This was reversible by including dithiothreitol in the assay buffer suggesting the loss of activity was due to oxidative inhibition of the enzyme. The decrease seen in the HT-29 cells was not sufficient to mimic the phenotypic changes seen following gene deletion.

### Role of p53 in NAT1 knockout phenotype

**Fig 4:**
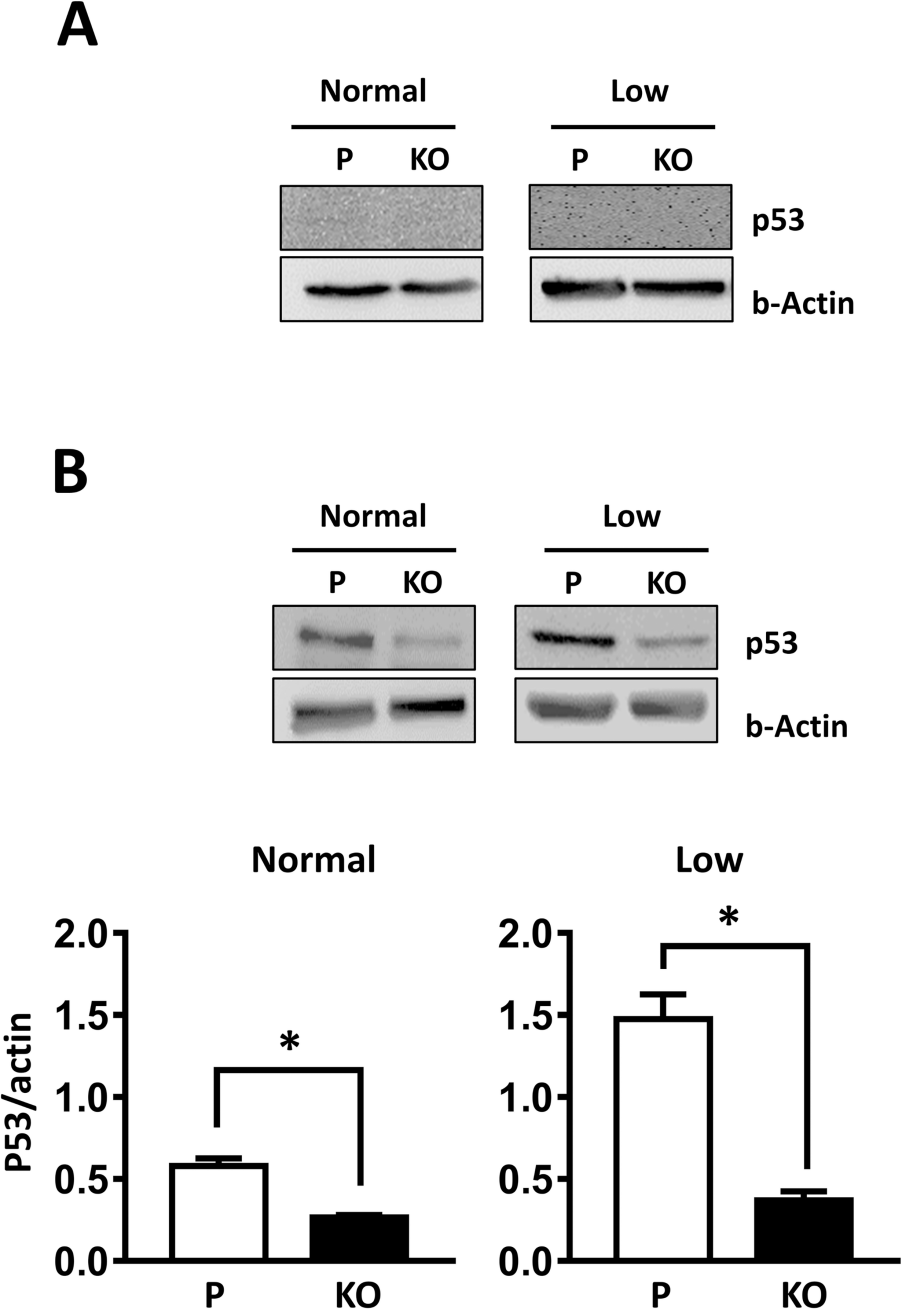
Changes in p53 expression following glucose starvation. (A) p53 expression in Parental (P) and NAT1 knockout (KO) HeLa cells grown for 24 hr in the presence of normal (10 mM) or low (1 mM) glucose. Blots are representative of 3 independent experiments. (B) p53 expression in Parental (P) and NAT1 knockout (KO) HT-29 cells grown for 24 h in the presence of normal (10 mM) or low (1 mM) glucose. Protein levels were quantified by densitometry and the data are mean ± sem, n = 3. Asterisk indicates significant difference by Student’s *t*-test.

HT-29 cells carry a gain-of-function p53 with a mutation (R^273^H) in the DNA binding domain of the protein. This causes an accumulation of p53 in these cells, which contributes to their oncogenic growth [17]. By contrast, HeLa cells express a wild type p53 that is down-regulated to low or undetectable levels [17]. This difference in p53 expression may contribute to the differences seen in the two cell-lines. To investigate this possibility, p53 levels were quantified in the HeLa (Fig 4A) and HT-29 (Fig 4B) cells following NAT1 knockout. There was no detectable expression of p53 in the HeLa cells either under normal glucose or low glucose conditions. By contrast, p53 was readily detectable in the HT-29 cells and glucose starvation increased p53 expression approximately 2-fold compared to that in cells grown in 10 mM glucose (Fig 4B). NAT1 knockout significantly decreased p53 both under normal glucose and glucose starvation conditions.

**Fig 5:**
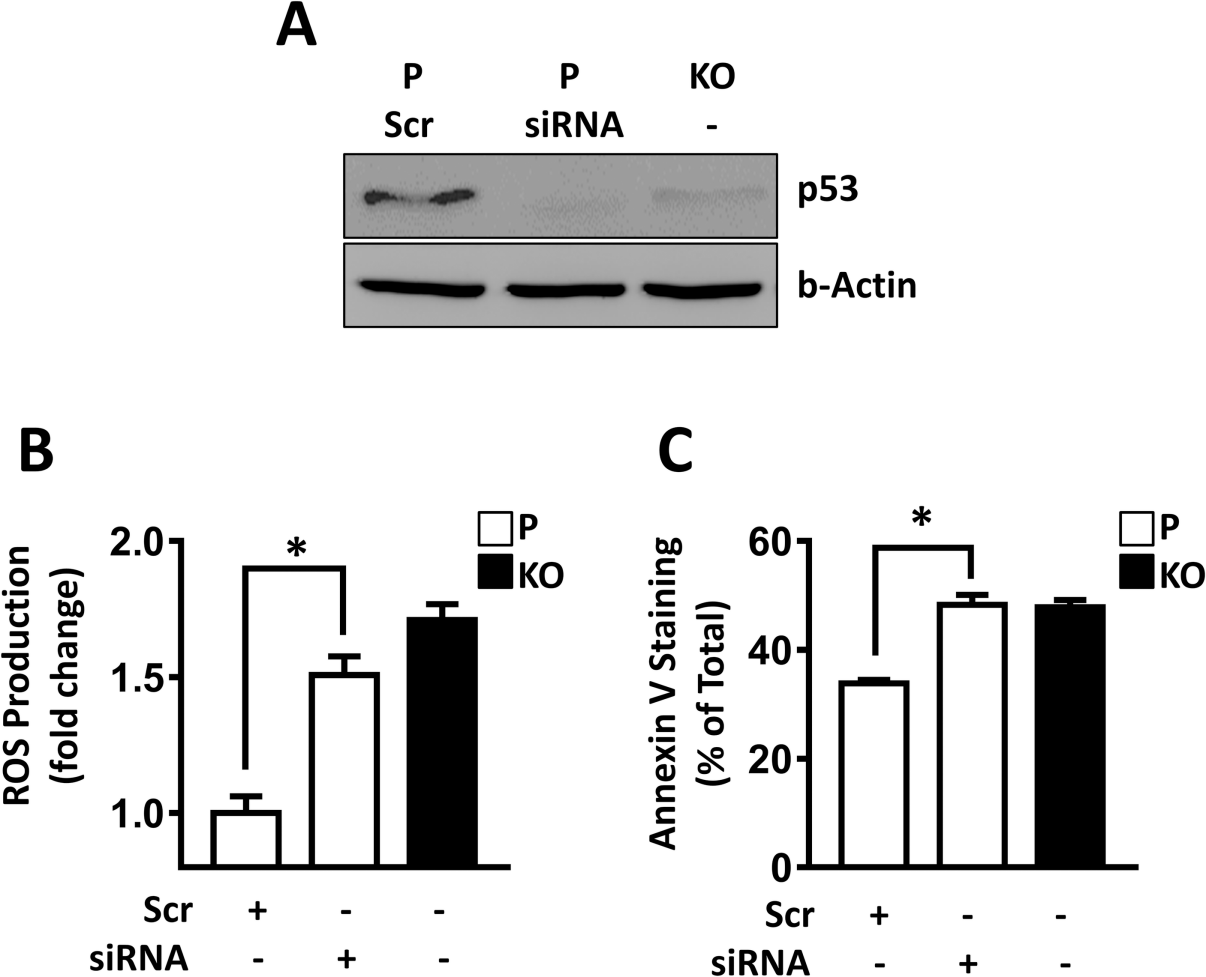
Effect of p53 siRNA on ROS production and cell apoptosis. (A) Parental HT-29 cells (P) were treated with scramble (Scr) or p53 siRNA for 72 hr and p53 protein was determined by Western blot. Untreated NAT1 knockout cells (KO) were also analyzed for comparison. (B) Effect of p53 siRNA on ROS production in Parental cells grown in low (1 mM) glucose. NAT1 knockout cells (closed bars) are shown for comparison. Data were expressed relative to Parental cells treated with scramble siRNA. (C) Effect of p53 siRNA on apoptosis in Parental cells grown in low (1 mM) glucose. Data are mean ± sem, n = 3. Asterisk indicates significant difference by one-way ANOVA.

To determine whether the loss in p53 seen in the HT-29 cells could account for the increase in ROS following NAT1 deletion, HT-29 parental cells were treated with p53 siRNA and ROS were measured following growth in low glucose (Fig 5). siRNA treatment decreased p53 levels to less than that seen following NAT1 knockout (Fig 5A). Moreover, it significantly increased ROS production to that seen in the NAT1 deleted cells (Fig 5B). Finally, p53 knockdown increased apoptosis in the parental cells (Fig 5C). Taken together, these results show that NAT1 deletion inhibited p53 expression in HT-29 cells, and this was responsible for the increased cell death seen during glucose starvation.

## Discussion

Although NAT1 is a drug metabolizing enzyme, recent studies have shown that it participates in a number of biochemical pathways involved in cell metabolism. Specifically, NAT1 can hydrolyze acetyl coenzyme A in a folate-dependent manner [18, 19] and it is important for palmitoleic acid homeostasis [20] as well as the methionine salvage pathway [4]. Here, the effects of NAT1 on cell survival during glucose starvation are reported for the first time. The results suggest that NAT1 has a role in the maintenance of gain-of-function p53, which attenuates ROS production during glucose starvation to promote tumor cell survival.

ROS-dependent oxidative stress following glucose starvation is well documented [21, 22]. ROS can be generated by NADPH oxidase activation or through mitochondrial respiration [23]. Moderate intracellular ROS levels are important in tumor cells and have been associated with numerous signaling pathways [24]. However, high intracellular ROS production can induce apoptosis [25]. Gain-of-function p53 can moderate ROS levels by up-regulating a variety of anti-oxidant genes. Moreover, it can influence glycolysis through the induction of TIGAR, which enhances glucose flux through the pentose phosphate pathway to increase NADPH, a cofactor in many anti-oxidant processes [25].

**Fig 6:**
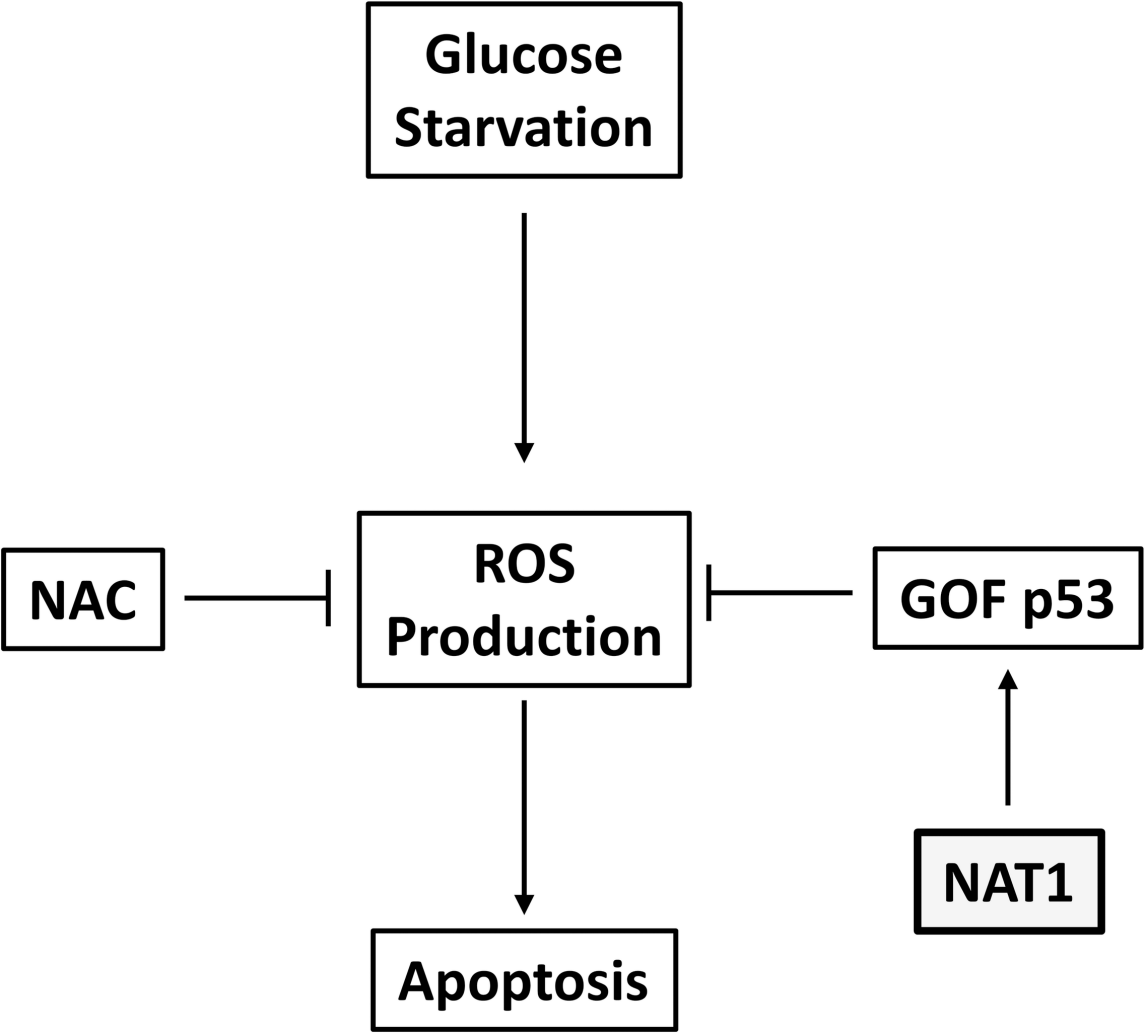
Schematic representation of the possible role for NAT1 in the regulation of ROS production and apoptosis. Glucose starvation increases ROS production which, at sufficiently high levels, will trigger apoptosis. The glutathione precursor N-acetylcysteine (NAC) prevents elevated ROS protecting the cells from oxidative damage. Glucose starvation also increases gain-of-function (GOF) p53, possibly via the AMPK pathway. The elevated p53 can attenuate intracellular ROS production protecting cells from oxidative stress. NAT1 increases GOF p53 and attenuates ROS production, although the exact mechanism for this effect is unknown. Consequently, deletion of NAT1 will increase intracellular oxidative stress. This model suggests NAT1 may be a target to decrease intracellular GOF p53.

The relationship between glucose starvation, ROS production and p53 is depicted in Fig 6. From the results in the current study, NAT1 can be added to this pathway as it is essential for the regulation of ROS by p53, at least in HT-29 cells. How NAT1 might influence p53 stability remains unknown. p53 is usually degraded via polyubiquitination in a Mdm2-dependent manner [26]. Thus, up-regulation of Mdm2 following NAT1 deletion may result in a decrease in p53 protein. Many of the ubiquitination sites on p53 are also acetylated by acetyltransferases such as p300/CBP, PCAF and Tip60, and deacetylated by Sirtuin 1 [26]. A change in p53 acetylation may also affect its stability.

There are 6 hot-spot residues in the DNA binding domain of p53 that account for many of the mutations observed in human cancers. Some of these result in changes in p53 binding to DNA while others result in structural changes to the protein. HT-29 cells express high levels of the R^273^H mutation, which changes DNA binding. The gain-of-function p53 found in HT-29 cells was up-regulated by glucose starvation (Fig 4B). Knockout of NAT1 almost completely down-regulated p53 indicating that targeting NAT1 might be a useful strategy in tumors driven by gain-of-function p53. These results help explain previous studies on the effect of NAT1 inhibition on cancer cell growth and invasiveness. In both HT-29 cells and MDA-MB-231 cells, a decrease in NAT1 expression significantly inhibits tumor growth in mice [3, 7]. Indeed, in the MDA-MB-231 cells, metastasis to the lungs was reduced by 95%. Both of these cell-lines express mutant p53 (R^273^H and R^282^H, respectively). NAT1 activity in the host also may be important for tumor progression. In support of this, a recent study by Stepp et al [27] demonstrated in rats that rapid acetylator animals develop chemically-induced tumors significantly more quickly than congenic slow acetylator animals.

In HeLa cells, glucose starvation decreased growth and increased apoptosis. However, NAT1 knockout had no effect on either of these measurements. HeLa cells express wild type p53, which was not detectable even following glucose starvation. These cells also harbor the papillomavirus HPV18 and express E6, a viral protein that promotes p53 degradation [28]. The lack of effect of NAT1 deletion in HeLa cells is consistent with it attenuating gain-of-function p53 to increase ROS production and apoptosis during glucose deprivation. It also suggests that NAT1 deletion may be innocuous to cells without mutant p53.

This is the first study that links NAT1 expression with p53 function, although these results need to be verified in additional cell models. Of importance is whether other mutp53 proteins are affected by the down-regulation of NAT1. Currently, there is no underlying mechanism that explains the results reported here. Nevertheless, they provide a foundation for future experiments to identify the biological significance of NAT1 in human cells. The development of CRISPR/Cas9 cell models will be an invaluable tool for these studies.

In summary, the current study has established a novel role for NAT1 in cancer cells, especially during nutrient-deprived conditions. These findings may be physiologically relevant because NAT1 is genetically polymorphic with large inter-individual variation [2]. Moreover, many solid tumors express gain-of-function p53 and are nutrient deprived due to poor blood supply. It is now important to identify the molecular pathway that links NAT1 expression with p53 activity/stability.

## Supporting information

S1 Fig Evidence for NAT1 knockout. A. PCR of genomic DNA isolated from HeLa and HT29 NAT1 knockout cells. PCR was performed using a forward primer in the NAT1 5’UTR and a reverse primer in the CRISPR insert to produce a product of ~ 800 bp.Parental HeLa cells are shown on the left as a negative control. B. Western blot of NAT1 expression in parental (P) and knockout (KO) HeLa and HT29 cells showing the absence of protein following CRISPR/CAS9 mediated gene deletion.
(PDF)
