# Supplementary material for: Arylamine N-acetyltransferase 1 protects against reactive oxygen species during glucose starvation: Role in the regulation of p53 stability
Source: PLoS One. 2018 Mar 8;13(3):e0193560. doi: 10.1371/journal.pone.0193560 (PMC5843258; doi:10.1371/journal.pone.0193560)
Supplement: S1 Fig — A. PCR of genomic DNA isolated from HeLa and HT29 NAT1 knockout cells. PCR was performed using a forward primer in the NAT1 5’UTR and a reverse primer in the CRISPR insert to produce a product of ~ 800 bp.Parental HeLa cells are shown on the left as a negative control. B. Western blot of NAT1 expression in parental (P) and knockout (KO) HeLa and HT29 cells showing the absence of protein following CRISPR/CAS9 mediated gene deletion. (PDF) [file pone.0193560.s001.pdf]

Supplementary Figure 1

A

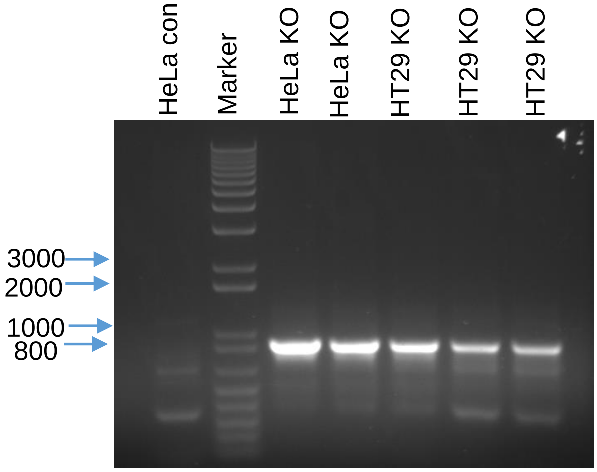

B

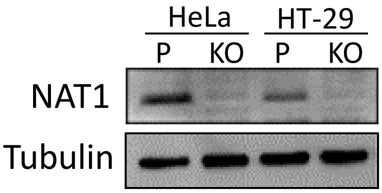

Fig S1 – Evidence for NAT1 knockout. A. PCR of genomic DNA isolated from HeLa and HT29 NAT1 knockout cells. PCR was performed using a forward primer in the NAT1 5'UTR and a reverse primer in the CRISPR insert to produce a product of ~ 800 bp. Parental HeLa cells are shown on the left as a negative control. B. Western blot of NAT1 expression in parental (P) and knockout (KO) HeLa and HT29 cells showing the absence of protein following CRISPR/CAS9 mediated gene deletion.
